# ‘Better sleep, better wellbeing’: Qualitative process evaluation of a hybrid, digital cognitive behavioural therapy programme for employees with sleep and emotion regulation problems

**DOI:** 10.1111/bjhp.70041

**Published:** 2025-12-04

**Authors:** Anna Hurley‐Wallace, Sophie Fletcher, Talar R. Moukhtarian, Krishane Patel, Agatha Payne, Tabitha Jackson, Carla Toro, Guy Daly, Sean Russel, Lukasz Walasek, Nicole K. Y. Tang, Caroline Meyer

**Affiliations:** ^1^ Musculoskeletal Research Unit, Bristol Medical School University of Bristol Bristol UK; ^2^ Division of Health Sciences, Mental Health and Wellbeing Group, Warwick Medical School University of Warwick Coventry UK; ^3^ Natwest Group London UK; ^4^ Department of Psychology, Clinical and Applied Psychology Unit University of Sheffield Sheffield UK; ^5^ Centre for Applied Psychology (CAP), School of Psychology University of Birmingham Birmingham UK; ^6^ The British University in Egypt Cairo Egypt; ^7^ Faculty of Health and Life Sciences Coventry University Coventry UK; ^8^ Department of Psychology University of Warwick Coventry UK

**Keywords:** CBT‐I, digital intervention, insomnia, mental health, process evaluation, thematic analysis

## Abstract

**Introduction:**

Sleep and mental health problems are common across the working adult population. This process evaluation provides insight into the experiences of employees who took part in a digital intervention trial: Supporting Employees with Insomnia and Emotional Regulation Problems (SLEEP). The programme combined digital CBT for insomnia with emotion regulation. Digital content was supported by remote therapy. The objectives of this process evaluation were to explore participants' experiences of the intervention, and identify how the intervention achieved change.

**Methods:**

Twenty‐one semi‐structured interviews were conducted using videoconferencing. A stratified sample of participants from within each of five cohorts of the SLEEP trial was interviewed. Thematic analysis utilized a collaborative codebook and framework approach. To conceptualize mechanisms of change, behaviour change techniques were retrospectively coded onto participant interview data.

**Results:**

An overarching theme: ‘Better sleep, better wellbeing’ was generated, with three interlinking themes conceptualizing the process by which positive changes to sleep and wellbeing were achieved. These were: ‘Procedure: The value of therapy sessions versus digital‐only’, ‘Context: Working on mental health from home during COVID‐19’, and ‘Mechanisms: Practice, feedback and problem solving.’

**Conclusions:**

Participants' experiences of SLEEP were predominantly positive and suggested a spillover effect of improved sleep on overall wellbeing. Triangulation of quantitative outcomes showed congruent improvements. Maintaining therapist contact to facilitate behaviour change throughout the programme was important. Furthermore, providing a private space for therapist calls was essential to facilitate the intervention in the workplace; an important insight for the development of digital mental health interventions intended for the workplace.

**Statement of Contribution**

**What is already known on this subject?**

- Sleep disturbances are prevalent among working adults, impacting wellbeing and workplace productivity.
- Digital CBT for insomnia can improve sleep and wellbeing outcomes for employees.

**What does this study add?**

- Integrating digital CBT for insomnia with emotion regulation can enhance sleep and wellbeing in working adults.
- Therapist support facilitates behaviour change in digital interventions targeting sleep and wellbeing outcomes.
- Providing a private space for therapy calls is crucial for workplace‐based digital mental health interventions.

## INTRODUCTION

Insomnia can be described as dissatisfaction with sleep quantity or quality, manifested as difficulty initiating or maintaining sleep, which is present on at least three nights per week for a minimum of 3 months (American Psychiatric Association, 2013; Morin & Benca, 2012). Insomnia disorder has an estimated global prevalence of 12.4% (van Straten et al., 2025). Further, symptoms of insomnia are reported by 30–48% of adults worldwide, based on general population studies conducted in countries including the UK, Europe, the USA, and Japan (Ohayon, 2002; Stewart et al., 2006). In the UK specifically, data from the second National Survey of Psychiatric Morbidity reported that 37% of adults living in England, Wales, and Scotland experience insomnia symptoms (Stewart et al., 2006). These symptoms can impact social, psychological, and occupational functioning (Espie et al., 2012; Yang et al., 2018). Furthermore, poor sleep is associated with decreased productivity and reduced work satisfaction (Daley et al., 2009; Kucharczyk et al., 2012). The number of working people experiencing symptoms of insomnia presents a major public health concern. Furthermore, Research AND Development (RAND) Europe reports that one in three workers in the UK is impacted by sleep problems, and due to productivity loss, this costs the UK economy an estimated £36 billion per annum (Hafner et al., 2016).

Cognitive behavioural therapy (CBT) is the recommended first course of treatment for individuals with insomnia (Edinger et al., 2021; Riemann et al., 2017). Meta‐analyses have estimated a moderate to large positive effect of CBT for insomnia (CBT‐I) on sleep quality and sleep efficiency (Wu et al., 2015). Delivering CBT‐I via workplace settings has the potential to improve the cost‐effectiveness of treatment delivery (Zachariae et al., 2016). Furthermore, evidence suggests that interventions targeting improved sleep for employees have the potential to improve productivity (Redeker et al., 2019), thereby reducing the economic impact of sleep problems (Hafner et al., 2016).

Despite indications of potential benefit to employers and the wider economy, there are a limited number of workplace sleep interventions that have been trialled. A systematic review of workplace insomnia interventions (Vega‐Escaño et al., 2020) identified two studies that have investigated the effectiveness of CBT‐I for employees (Bostock et al., 2016; Suzuki et al., 2008), of which only one yielded significant improvements in work productivity (Bostock et al., 2016). There is scope here for the development of novel interventions to support employees in improving their sleep. Further, digitization may help streamline the delivery of CBT‐I, resulting in easier access to sleep‐specific support, with potential cost benefits. Analyses of digitized CBT found cost savings of between £116 and£136 million per annum in England compared with face‐to‐face therapy (Improving Access to Psychological Therapies (IAPT) Programme, 2007).

### Supporting employees with insomnia and emotional regulation problems (SLEEP)

SLEEP is a hybrid, digital CBT‐I + emotion regulation (dCBT‐I + ER) intervention, one of six interventions targeting improvements to employee mental health and productivity across the Midlands, UK (https://midlandsengine.org/mental‐health‐productivity‐pilot/). The SLEEP programme duration was 6 weeks, inclusive of digital content and four one‐to‐one videoconferencing therapy sessions with a trained CBT‐I therapist. All therapists undertook a 3‐day training course (7 sessions) in CBT‐I, delivered by Universitätsklinikum Freiburg Centre for Mental Disorders and led by Professor D Riemann (April 2021). Therapists were matched with participants depending on availability.

Participants were recommended to spend ~ 1 hour per week working through digital content on the intervention platform and were asked to complete a sleep diary throughout the entire programme. The CBT‐I component of SLEEP utilizes behavioural, cognitive, and psychoeducation techniques to address sleep problems (Edinger et al., 2021; Riemann et al., 2017), and the ER component consists of relaxation techniques as well as practical tools to improve stress and worry management (Berking et al., 2008). ER content was included in the programme because sleep problems are closely related to difficulties people can experience in regulating their emotions (Van Someren, 2021); people with insomnia are five times more likely to report anxiety and depression symptoms (Pearson et al., 2006).

The SLEEP intervention has been trialled using a randomized wait‐list control design (Moukhtarian et al., 2025). The main RCT presenting the quantitative outcomes of this trial, including effects on primary outcomes (insomnia severity, depression and anxiety symptoms), and secondary outcomes (wellbeing, quality of life, job satisfaction, and productivity) is reported elsewhere (Moukhtarian et al., 2025). Adding to the interpretation of trial outcomes, process evaluations can be utilized to explore the mechanisms and context through which interventions achieve change. This is particularly useful for understanding the generalisability of effectiveness to other populations and contexts (Moore et al., 2015). The process evaluation reported herein seeks to understand participant experiences of the intervention, including psychological and behavioural processes underpinning these experiences, and contextual factors. These qualitative findings are then triangulated with quantitative findings from the main trial, per guidance (Moore et al., 2015), to better understand how outcomes were achieved.

The main objective of this qualitative process evaluation was to: explore participants' experiences of the SLEEP intervention, including (i) identifying and exploring facilitators and barriers to engagement, (ii) identifying behaviour change mechanisms (Michie et al., 2013), and (iii) exploring to what extent contextual factors may have impacted engagement and subsequent outcomes of the intervention.

## METHODS

The process evaluation report follows the COREQ reporting criteria for qualitative research (Tong et al., 2007). A protocol providing detailed methodology was published ahead of analysis (Hurley‐Wallace et al., 2022). This study used semi‐structured, individual interviews conducted via online videoconference (Microsoft Teams). The interview schedule, consisting of eight questions (and several prompts) is provided as a supplement to this report (Data S1). The interview schedule was pre‐piloted between members of the research team. Data analysis used codebook style thematic and framework analysis (Braun et al., 2019; Braun & Clarke, 2021a; Ritchie & Spencer, 2002).

### Patient and public involvement

This process evaluation is part of the SLEEP study, which sourced patient and public involvement (PPI) input for its design and implementation. This is described elsewhere (Moukhtarian et al., 2025). The process evaluation interview schedule did not receive specific comments from PPI contributors.

### Research team characteristics

The team of researchers conducting interviews and involved in subsequent qualitative coding and analyses consisted of six researchers with academic and/or clinical backgrounds in psychology. Three members of the team hold PhDs (AHW, KP, and TM) and three members of the team hold master's‐level qualifications in psychology (ST, AP, and TJ). Qualitative analyses were led by AHW, who specializes in health psychology, and took a critical realist epistemological stance to analyses described in the current report.

Two members of the interviewing/coding team had no prior relation to the study participants (AP, TJ). Four members of the research team (AHW, ST, KP and TM) worked as the four remote therapists who delivered the therapy sessions for the SLEEP intervention. All four therapists undertook a 3‐day training course (7 sessions) in CBT‐I, delivered by Universitätsklinikum Freiburg Centre for Mental Disorders and led by Professor D Riemann (April 2021). They were also supervised by a Clinical and Health Psychologist with expertise in CBT‐I (NT) throughout the SLEEP trial. Therapists did not interview participants they worked with during the programme. Mobilizing these therapists as members of the interviewing/coding team helped to streamline the analytical process and better understand interviewees' experiences, as these team members have a detailed understanding of the hybrid treatment content and delivery context.

### Sample size and saturation

A stratified sample of 21 participants was obtained across five cohorts from the main SLEEP intervention trial (Moukhtarian et al., 2025).

The initial target sample size for this process evaluation was 25 interviews (Hurley‐Wallace et al., 2022). Research on saturation in thematic analysis indicates saturation of meaning approximates 16–24 interviews and represents an in‐depth understanding of identified issues (Hennink et al., 2016). However, within thematic analysis new meanings are always theoretically possible (Braun & Clarke, 2019; Low, 2019). The research team interpreted, through team discussion, that high saturation of meaning was achieved based on interview data from 21 participants.

### Participant selection

A total of 35 participants were invited to participate, where 14 did not respond. Zero participants formally declined; hence no reasons for non‐response were given.

Stratification aimed for an equal weighting of participants between the treatment and wait‐list control arms of the RCT (Moukhtarian et al., 2025). Participants were randomly selected to be invited from within each cohort. Only participants who consented to be contacted for a post‐intervention interview were invited, by email, to take part. If there was no response, a follow‐up email was sent 1 week later. No further contact was made if a reply was not received. Where participants were approached and did not respond, another participant was randomly selected from those remaining within the same cohort.

It is worth noting that participants involved in the interviews, as well as the wider trial, were primarily employed in office‐based or hybrid roles across sectors including education, IT, manufacturing, information and communication, business administration and support services, and retail. Most had the flexibility to work from home for some or all of their working week. Another important context from the main trial is participant therapy sessions. Participants from the main trial had contact with the same therapist as much as possible (four, 1‐h sessions per participant). Therapy session scheduling was done pragmatically based on the time availability of participants and therapists. It was not always possible for participants to have the same therapist for all four sessions throughout the intervention. On these occasions, another therapist would stand in following a handover. Some participants from the trial, and subsequently the evaluation interviews, had contact with 2 or 3 therapists.

Recruitment of participants for process evaluation interviews commenced from 15 September 2021 (first invites) until 29 March 2022. Recruitment for the main SLEEP trial commenced on 18 June 2021 until 31 October 2021.

### Ethical approval and consent

Full approval for the SLEEP study, including qualitative process evaluation interviews, was given by the University of Warwick Biomedical and Research Ethics Committee (BSREC 45/20‐21). Qualitative interviews required a written and verbal consent form (in addition to the consent form from the main SLEEP trial). Consent forms were signed electronically by each interviewee and returned by email prior to attending the interview. Verbal consent was additionally recorded at the beginning of each interview.

### Interview procedure

Interviewees were allocated an interviewer from the research team, who was independent from the interviewee's treatment delivery team (i.e., participants were not interviewed by their SLEEP therapist). This was done to ensure the confidentiality of responses, as well as encourage the expression of honest opinions. Participants were invited to attend an online videoconference meeting via Microsoft Teams at a mutually agreed time.

Interviews were conducted 1:1 with no additional team members, chaperones, or interpreters present. Each interview process began with a standardized introduction. Audio recording was then started and verbal consent was obtained. The interview then commenced, following a semi‐structured, open‐ended schedule (Data S1). Interviews lasted for a mean length of 27 min (range: 15–40 min). Participants were given an opportunity to ask any questions once the interview had finished and the recording had been stopped.

#### Recording

OBS studio was used to record the audio‐only consent and interview data (obsproject.com). Microsoft Teams was not used to record interviews, as there was no option to record audio‐only, and video was not necessary. No interviews were repeated; however, one interview did not record successfully; hence, it was not included in the analysis or numbers presented.

#### Transcription

Audio recordings were initially saved in an access‐restricted, secure digital folder within the University of Warwick servers. They were then sent to a third‐party University of Warwick‐approved vendor to be transcribed verbatim. Transcriptions were anonymized by the external provider (removal of names, locations and identifying characteristics), and were not returned to participants for comments or correction, due to study timeline constraints.

#### Data protection

All data collection and analysis were compliant with the General Data Protection Regulation (GDPR) and the Data Protection Act 2018. All data for this study were electronic only and were stored in digitally secure folders within the University of Warwick, with restricted access (password‐protected) while analysis was ongoing.

Audio recordings were deleted upon trial completion (ISRCTN13596153). Pseudonymized transcriptions and study consent forms will be stored on the University of Warwick shared secure servers for 10 years as per the University of Warwick regulations for data usage and storage.

### Qualitative analysis

#### Methodological approach

The approach to qualitative analysis combined codebook‐style thematic and framework analysis (Braun et al., 2019; Braun & Clarke, 2021a; Ritchie & Spencer, 2002). The analysis was inductive, using a data‐driven approach to develop themes. A flowchart of the analytic approach, as well as a detailed rationale for selecting this approach is provided in the study protocol (Hurley‐Wallace et al., 2022). A major benefit of combining codebook thematic and framework analysis is that this allows for the underpinning foundation of the analysis to remain interpretative (Braun et al., 2019; Braun & Clarke, 2021b), whilst simultaneously creating a clear structure for the qualitative codebook. Applying a framework to the codebook in the early stages helped retain focus on the objectives of the process evaluation (Braun & Clarke, 2021b), whilst working as a collaborative group of six coders, with varying levels of qualitative experience.

#### Analytic procedure

Adhering to the six phases of thematic analysis (Braun & Clarke, 2006), interview transcripts were initially reviewed by each interviewer whereby audio‐recordings were cross‐checked against transcriptions. Interview transcripts were then imported to NVivo (QSR International Pty Ltd, 2012).

Initial qualitative coding of the first six transcriptions was completed by each researcher that conducted the interview in question. Initial coding was done in units of meaning (i.e., a phrase to a paragraph), and some coding overlap (up to three codes) was allowed. Following coding of the first six interviews, the interviewers met to discuss their interpretations and exchange ideas.

The lead researcher (AHW) then collated the inductive codes from the first six interviews into a codebook, to which a framework was applied. To begin with, the framework was structured based on the research objectives (i.e., participants' experiences of the intervention; barriers and facilitators to engagement, contextual factors impacting change; Parkinson et al., 2016). This first framework was applied to one interview at a time; transcripts and coding from each interview were imported into the version of the NVivo project containing the framework. As each new interview was added in, codes were combined and iterated as needed to avoid duplication of codes with the same underlying meaning. Once the first six interviews were integrated, the qualitative team (including all six interviewers) then met to discuss and make a consensus agreement on the initial codebook and framework. Team discussions were held weekly to bi‐weekly whilst the qualitative analysis was ongoing.

The remaining interview transcripts were integrated in chronological order of interview date, using the codebook and framework as a guide. At this stage, interviews could be coded by any member of the qualitative team, and new codes could be added as needed by any team member. The framework was also iterated by AHW, with consensus agreement from the qualitative team, throughout coding. This resulted in one additional framework category, ‘content suggestions’, in addition to the originally defined categories: overall experience; barriers and facilitators; behaviour change mechanisms; and contextual factors. The finalized codebook, with the framework applied, is available with this report (Data S2).

Once all interview data had been initially coded, and the codebook and framework were finalized, the qualitative team met to discuss ideas for themes. AHW and ST, having coded the greatest proportion of interviews within the team, then created individual thematic maps. AHW, ST, KP, and TM then met again to discuss the two sets of themes. The themes were then collated together by AHW. Lastly, the thematic map was triangulated and iterated with input from the SLEEP lead clinical psychologist (NT). Participants did not provide feedback on the findings from the thematic analysis, due to study timeline constraints.

#### Identifying behaviour change mechanisms

To gain an understanding of the underlying behavioural mechanisms that may have impacted intervention outcomes (i.e., changes to sleep and emotional regulation), Behaviour Change Techniques (BCTs) were coded onto participant interview data, consulting the BCT Taxonomy (v1) (Michie et al., 2013). BCTs were coded retrospectively (rather than representing an a priori defined underpinning framework), and then mapped into the developing thematic analysis. The top four most commonly coded BCTs are presented as mechanisms of change within the thematic analysis, with agreement from the analytical team that these mechanisms had the most noticeable impact across the group of participants, achieving saturation across >50% of the interview sample (all BCT codes are available in the codebook; Data S2).

## RESULTS

Twenty‐one interviews were successfully recorded for this study, representing a 15% proportionate uptake from the trial sample (*n* = 136). One interview did not record and therefore could not be included in the analysis. The interviewee group consisted of participants from treatment and wait‐list control groups, as all participants received the intervention, with outcomes evaluated at different time points. A demographic summary of the interview sample is provided in Table 1.

**TABLE 1:** Demographic summary of interviewees (*n* = 21).

	Wait‐list control (*n* = 8)	Treatment (*n* = 13)
Age (M, SD)	39.75 (9.77)	46.52 (8.71)
Gender (*n*, %)		
Female	5 (63%)	8 (62%)
Male	3 (32%)	5 (38%)
Ethnicity (*n*, %)		
Asian	0 (0%)	3 (23%)
Black	0 (0%)	1 (8%)
Other	1 (13%)	0 (0%)
White	7 (88%)	9 (69%)
Income (*n*, %)		
£10,000–£29,000	1 (13%)	2 (15%)
£30,000–£49,999	5 (63%)	3 (23%)
£50,000–£69,999	0 (0%)	4 (31%)
£70,000–£89,999	2 (25%)	2 (15%)
£90,000–£109,999	0 (0%)	2 (15%)

### Thematic analysis

**FIGURE 1:**
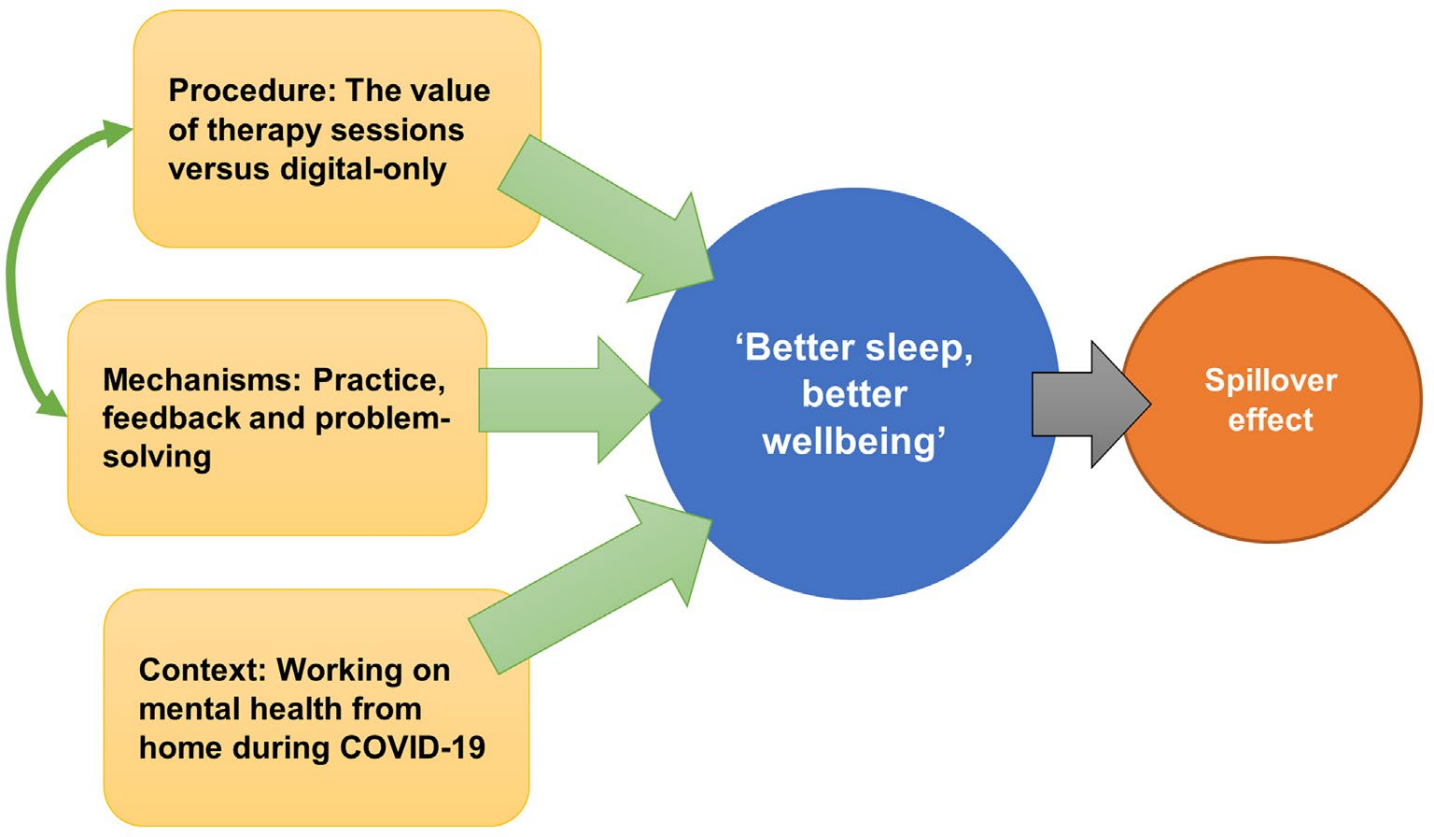
Process evaluation thematic map for the SLEEP trial.

An overarching theme: ‘Better sleep, better wellbeing’ was generated, with three interlinking themes conceptualizing the process components by which positive changes to sleep and wellbeing were achieved. These are displayed in a thematic map (Figure 1).

The integrated map provides a dynamic picture of the conceptualized active components of change within the SLEEP intervention (mechanisms and procedure, which are interlinked), and contextual drivers of change that may have influenced outcomes (i.e., COVID‐19 context).

According to participant accounts, experiences of hybrid dCBT‐I + ER were predominantly positive and suggested an impact of improved sleep on overall wellbeing, as well as a spillover effect beyond targeted outcomes (sleep, mood [anxiety, depression], and work‐related outcomes; Moukhtarian et al., 2025). Therefore, ‘Better sleep, better wellbeing’ was generated as an overarching, outcome‐oriented theme. ‘Spillover effect’, also known as behavioural spillover (Dolan & Galizzi, 2015), is a subtheme of ‘Better sleep…’ and indicates the impact of the intervention beyond the outcomes targeted in the trial. For example, improved sleep inherently elicited improvements in work‐life balance and relationship dynamics.

To conceptualize how these overall positive outcomes were achieved, three component themes were generated. First, the intervention procedure; maintaining structured therapeutic contact throughout the digital programme was vital in the success of SLEEP. Participant experiences emphasized the added value of therapist sessions within the digital intervention, where tailoring advice to individual needs (tailored problem‐solving) increased engagement and subsequently improved outcomes. Second, behaviour change mechanisms were identified within participant responses, alluding to how participants were able to achieve positive changes in their sleep, for example, through behavioural practice. Third, COVID‐19 was identified as a contextual driver of change. Having a private space to take therapy session calls was important to employees, and indeed the ‘working from home’ period in 2021 is likely to have enhanced engagement with the intervention, because of the available privacy compared with in‐office working.

It is important to note that not all experiences were positive, where there was one negative account of a poor therapist match for the participant, with the statement that the programme was less helpful than other (private) therapy that they had received. The participant described that they ‘felt like a data point’, which may reflect the protocolized nature of the intervention in the context of a controlled trial. Although there were tailored aspects of SLEEP, therapists in this trial could not include components from other types of psychological therapy, despite the fact that further tailoring may have been helpful for some participants. There were some other comments by participants regarding therapist matching or ‘therapeutic alliance’. These were predominantly positive or neutral in nature, although some participants highlighted that they would have preferred the same therapist across all four sessions offered. This was not always possible as matching depended upon mutual availability.

#### ‘Better sleep, better wellbeing’

This overarching theme is outcome‐oriented, reflecting that most participants who were interviewed perceived positive outcomes in the areas of sleep and wellbeing. This is important given the focus of the SLEEP programme content was on improvements in insomnia (ISI; Bastien et al., 2001), as well as secondary measures from participant sleep diaries (sleep efficiency and sleep quality scores).

Participant quotes exemplify the overall participant perception of positive outcomes achieved. These quotes vary from person to person as the intervention was hybrid in nature, and therapy was hence tailored to each participant's needs. For example, whilst all participant sleep diaries were monitored and sleep routines were updated throughout sessions 1–4, some participants would have focused more on ER versus CBT‐I techniques.

It was overall positive and I found it beneficial. I found that I… understood my sleep better as a result of that. And it also helped me with some issues that were impacting my sleep as well. (Participant A)

It was great. It's really helped me to step back from sleep issues and just to take better care of myself at the time. (Participant B)

There was also feedback from participants about the relevance of the programme across a wide variety of clientele, and references to how the programme should be shared with everyone, and that it should be endorsed by employers.

Really good, really relevant to everyday life, to cover quite a diverse set of individuals that will be using it. I thought it could be relevant to a lot of different people. It was certainly relevant to me. (Participant C)

I think it's a really, really important area to be looking at and I think to be able to roll out to more people in the‐ well, actually the [organisation] but for any employer would be a really good thing. (Participant D)

The importance of focus on sleep was also touched upon, particularly the idea that it is difficult to realize impact of sleep on wellbeing, until you start sleeping well.

I think it's just realising that these things are important. So, I wouldn't really consider that getting a good night's sleep was important as I've realised it was, once I started getting a good night's sleep. (Participant E)

#### ‘Spillover effect’

The above quote from participant E touches on the idea of a spillover or knock‐on effect of improved sleep on daily quality of life. For some of the accounts that pertain to general wellbeing improvements, it is impossible to disentangle how much of the improvement in wellbeing is due to additionally targeting emotional regulation within the SLEEP programme, or whether this effect is rooted in better sleep. For example:

I think it's made really important improvements to my sleep patterns and my quality of life and wellbeing in general so I really had highly positive views on the experience (Participant F)

For others, there was a clear‐cut distinction where participants felt their overall wellbeing had improved because they felt ‘fresh’ during the day, or that they had more energy to manage their busy working lifestyle.

I'd say work‐life balance, it has improved. Because now I'm getting more sleep, I feel like I'm not as worked up when I do go to work. And the fact that I feel more sort of energised in myself helps in terms of I want to do a bit more and I want to sort of… I'm more inclined to do like a bit of exercise and things like that if I'm not as worn out in the day. (Participant G)

I seem a bit…a lot calmer. (Pause) And a lot less sort of stressed or anxious at times. (Pause) And I think that was all just tiredness building up from disturbed sleep. (Participant H)

In relation to improvements in mood or ‘mental wellbeing’ (noting that anxiety and depression symptoms were targeted outcomes of the programme), several participants talked about the positive impact as surprising or exceeding expectations above and beyond solely improving sleep.

I think the one thing I hadn't considered that it would positively impact my mental wellbeing and that was – that happened more than I expected. So, that is a really nice kind of bonus. (Participant E)

I didn't think we would get in to the physical or mental wellbeing. And, oh my God (laughs). It did, and it was amazing. (Participant I)

Spillover effects may relate directly or indirectly to the ER aspects of the programme, which varied between participants as to how much input they wanted beyond addressing sleep problems (participant I). This level of tailoring was also part of what made the intervention successful.

#### Procedure: The value of therapy sessions versus digital‐only

Most of the SLEEP interviewees talked about the utility of the individual therapist sessions, and how this helped tailor what would have been relatively ‘generic’ content to their needs. Having the therapy session also provided an opportunity for clarification on content. Many participants talked about the sleep therapy sessions as the ‘best part’ of their experience.

I would say probably with the sleep therapist made the most difference. It was just sort of getting things in my head. So, when she was talking about little bits about the modules as well, it was just making things a little bit clearer. (Participant G)

Importantly, participants felt validated and supported throughout the programme, especially as many mentioned that engaging with sleep restriction therapy (SRT) was very hard at first. Perceived therapist competence was important in determining the sense of support that clients felt, with respect to listening skills and competence with sleep therapy content, particularly how to tailor content to individual needs.

I really like the non‐judgmental aspect of it because I'm a really late sleeper like a night owl type of person. And throughout my life, I always felt judged because of that. And there was no judgement whatsoever which I really liked. And especially during the therapy sessions, I felt the therapist really listened to me and my needs. (Participant F)

Accountability and alliance with the therapist were also important in terms of sticking to the programme. Not every therapy experience was positive, several were neutral in nature. However, what was uniform across the majority (except one negative experience aforementioned) was that having a therapy session booked in helped with accountability and thereby engagement with the programme, particularly sleep scheduling.

…regarding the set times going to bed and getting up each day. That was something that I've been thinking about doing for a long time, but I needed that push to get me to do it, and I found that really good. (Participant E)

#### Mechanisms: Practice, feedback, and problem‐solving

A theme conceptualizing behaviour change mechanisms that contributed to positive experiences and outcomes was conceptualized. We identified four BCTs (Michie et al., 2013) that mapped to greater than 50% of participant interviews. These were: (i) behavioural practice (BCT 8.1, *n* = 19), (ii) problem‐solving (BCT 1.2, *n* = 17), (iii) feedback on behaviour (BCT 2.2, *n* = 12), and (iv) reduction of negative emotions (BCT 11.2, *n* = 12). Given the programme content and integration of tailored therapist sessions, the BCTs identified are unsurprising. particularly as the SRT element of the programme required participants to keep a diary and practice a sleep schedule, as well as maintain routines using stimulus control therapy (SCT); this is behavioural practice in a nutshell.

I learned how to keep a constant, let's say, path and not change every night or following more or less sleep necessity but to respect the rules were useful. (Participant K)

Although intuitive, these insights remain helpful in streamlining future iterations of the SLEEP programme, as they reveal the underlying behavioural processes that appear to drive positive change in domains of sleep and mood. Problem‐solving was highlighted in the interview data as being directly related to therapist tailoring. For example:

Rather than it just being you followed this programme and then you're fixed. It was more sort of talking about it, tweaking it and fitting it into my lifestyle, rather than one‐size‐fits‐all sorts of thing (Participant E)

…it just gave you that kind of place to discuss things and get some help like kind of personalised to yourself rather than having something that might be a bit more generic to everybody, because I think if it was just the kind of materials that were provided and just tracking, I don't think I would've gotten as much out of it. (Participant L)

There was a high overlap of codes relating to problem‐solving and therapist tailoring. This connects the procedural theme ‘The value of therapy sessions versus digital‐only’ to the mechanisms theme ‘Practice, feedback and problem‐solving’. These components of the process evaluation are hence interpreted as interrelated.

Feedback on behaviour was also linked to the therapist sessions. The coding of BCT 8.1 (practice) and 1.2 (feedback) overlapped in several of the transcripts. A key difference is that the sleep diary was used as a feedback tool within the therapy sessions, helping to provide an important reflection on participant behaviour and how to change moving forward.

The sleep diary that we kept which you know, I mean there were a few sort of abnormalities or outliers if you like, in the amount of time, percentage time asleep, but it was‐ I think the qualitative interaction behind that was the most interesting… we had a discussion around that and that I found to be the most useful. (Participant D)

Reduction of negative emotions (BCT 11.2) was also integral within SLEEP, as use of ER techniques to facilitate improved sleep was part of the programme content. Interestingly, reduction in negative emotions sits as both a mechanism to facilitate better sleep, and a spillover effect from better sleep.

To emphasize the insights found on behavioural practice and feedback, and how these behavioural mechanisms worked within the SLEEP programme, in alliance with a therapist, a final quote from participant C exemplifies:

But what was nice was that actually during the sessions, it was evident that I was, my sleep pattern was better than I thought it was. And I also started to regularise my timings, which I hadn't been very strict at previously. It tended to improve it, so actually, the proof is in the pudding. (Participant C)

#### Context: Working on mental health from home during COVID‐19

An important contextual driver for engagement with the SLEEP programme was COVID‐19. In particular, the interview data suggested that the ‘working from home’ context improved engagement with the intervention, due to the digital nature of the whole programme. This included therapy calls delivered on Teams. It is likely that high engagement with the intervention impacted the positive outcomes achieved by the participants who were interviewed. This is supported by high completion rates for the online platform content (72.8%) (Moukhtarian et al., 2025).

There are two key points within this theme. First, it is evident that computer literacy has improved, in part because the setup and training to be able to complete the programme at home were provided to the employees.

I think we've all grown accustomed to doing so much of our lives online now that I didn't really have much of an issue with it. I don't think I could've done this any other way but online anyway (Participant M)

The Teams call that I did were also fine for me. I've got experience with Teams calls though because I seem to be doing them all the time at the moment, I think everyone is, aren't they? (Participant A)

Second, there were some qualitative codes that suggested participants' avoidance of bringing their mental health concerns to the workplace, with a preference for a separation of emotional and/or mental health issues from colleagues. Most participants talked about the ease of having a private space available at home, and a select few participants went further to highlight the potential challenge of becoming emotional at work.

I think it might have been more difficult to continue, not continue but to complete things in the same way when I'm in the office when you're physically visible to everybody. (Participant C)

I don't know whether it could be facilitated because if you do get emotional and you feel coming out of the meeting room emotional, you're not going to be able to – you don't get that privacy do you really, I suppose. (Participant N)

The above quotes further support that the COVID‐19 context was a key factor in the success of the SLEEP intervention. Beyond being physically visible, concerns about coming across as emotionally vulnerable at work are evident.

### Triangulation of qualitative and quantitative findings

Qualitative insights presented in the current paper warrant triangulation with quantitative outcomes from the main SLEEP trial paper (Moukhtarian et al., 2025), as part of complex intervention process evaluation. Triangulation was carried out post‐analysis of findings (Moore et al., 2015; O'Cathain et al., 2010). Findings were harmonious in this case, indicating congruent improvements in sleep and wellbeing across quantitative and qualitative analyses. Matching with the overall positive experiences deduced from the interview data, participants self‐reported that all quantitative scores for insomnia severity (ISI; Bastien et al., 2001), depression (PHQ‐9; Kroenke & Spitzer, 2002) and anxiety (GAD‐7; Spitzer et al., 2006) symptoms improved from baseline to follow‐up (week 8). Quantitative analyses using an intention‐to‐treat approach indicated significant time‐adjusted differences (*p* < .001) between the SLEEP intervention and wait‐list control group at week 8 (post‐intervention), with large effect sizes (ISI; *d* = 2.01, PHQ‐9; *d* = 1.79, GAD‐7; *d* = 1.39; Moukhtarian et al., 2025). Exploratory analyses were additionally conducted to evaluate treatment efficacy by comparing clinical caseness pre‐post study for both groups, using rates of clinically significant improvement and recovery in line with the Improving Access to Psychological Therapies (IAPT; now known as NHS Talking Therapies) framework (England, 2018). For the ISI, caseness is indicated by a score of ≥8, with a reduction of at least 8 points indicating clinically significant change (Morin et al., 2011). For the PHQ‐9, caseness is indicated by a score of ≥10, and for the GAD‐7 a score of ≥8. Clinically significant change is a reduction of 4 points on the GAD‐7 and 6 points on the PHQ‐9 (England, 2018). Clinically significant change was found for all three primary outcomes (Moukhtarian et al., 2025), hence participants in the intervention group who were categorized as having clinically significant depression, anxiety or insomnia, improved significantly over the course of the trial, compared with the control group.

Considering further triangulation of findings relating to participants' engagement with the intervention, the theme ‘Procedure: the value of therapy appointments…’ is supported by engagement statistics presented in the quantitative findings. Participants in the SLEEP intervention group completed on average 72.8% of the online content and attended an average of 3.4 (out of 4) therapy sessions (Moukhtarian et al., 2025).

The role of context is particularly important in the process evaluation of a trial such as SLEEP. Findings allude to the likelihood that the context of the COVID‐19 pandemic significantly enhanced engagement. Hence, whilst quantitative findings presented in the main trial paper for SLEEP lend support to the overall positive qualitative interpretation presented herein, effectiveness may be limited to a context in which many employees were working from home.

## DISCUSSION

The overarching theme from the qualitative evaluation of the SLEEP trial was ‘Better sleep, better wellbeing’, with a positive spillover effect to areas of life beyond sleep, mood, and work‐related outcomes (Figure 1). There were three component themes contributing to this predominantly positive intervention effect. Intervention procedures and mechanisms (BCTs) were interlinked, and the ‘working on mental health from home’ during the COVID‐19 context elicited an unexpected positive impact on engagement, leading to better participant outcomes.

Our findings align with previous evaluations of digital CBT‐I interventions, which similarly demonstrate that improvements in sleep are associated with benefits to work functioning and productivity in workplace settings (Bostock et al., 2016; Zettor et al., 2025). Improvements in mood and mental health symptoms have also been consistently evidenced across a recent systematic review and meta‐analysis of digital CBT‐I (Lee et al., 2023). However, the current study is distinctive in several important ways. Unlike many existing interventions that are fully self‐guided and focus solely on insomnia, the SLEEP programme integrates therapist‐led sessions with digital content in a hybrid delivery model, and uniquely incorporates targeted emotion regulation (ER) strategies. To the best of our knowledge, this is the first workplace‐based hybrid CBT‐I + ER intervention that combines digital modules with therapist support. This positions the current study as a novel contribution to both digital CBT‐I literature and workplace wellbeing programmes, especially in the context of scalable, flexible models suited to occupational settings.

Regarding behaviour change mechanisms, insights from participant interviews can help to streamline future iterations of the SLEEP programme, as well as other interventions that seek to target sleep and emotional regulation problems in a hybrid dCBT‐I + ER format. The BCTs (Michie et al., 2013) highlighted in the current qualitative analysis: behavioural practice, problem‐solving, feedback on behaviour, and reduction of negative emotions, can be used to envisage an underpinning logic model. As such, future interventions could increase focus on these behavioural aspects during early‐phase intervention design and development, to help achieve positive participant outcomes as have been evidenced in the SLEEP trial. For example, more problem‐solving exercises could be included, and therapist sessions could allocate more time to give behavioural feedback with reference to participant sleep diaries.

Within the ‘Mechanisms…’ theme it was identified that reduction in negative emotions using ER strategies can be conceptualized as a facilitating mechanism of improved sleep. However, reduction in negative emotions can also be a spillover effect resulting from improved sleep, as a direct effect of being less tired and having more energy to engage with activities of enjoyment. Participants described feeling calmer, less anxious, and more energized, either due to deliberate use of ER strategies introduced in the intervention or as a direct outcome of reduced sleep disturbance and daytime fatigue. This bidirectional relationship between sleep and ER supports emerging theoretical models that conceptualize these domains as interdependent systems, where improvements in one may reinforce and sustain gains in the other (Palmer & Alfano, 2017). However, it remains difficult to disentangle how much change in mood outcomes (depression and anxiety symptoms), seen in both the qualitative and quantitative (Moukhtarian et al., 2025) data from this trial, can be attributed to CBT‐I versus ER intervention components, particularly given the tailored nature of the therapist sessions, which varied in their ER content depending on individual needs.

Furthermore, from a behaviour change perspective, our findings suggest that hybrid digital interventions may support engagement and outcomes via two synergistic pathways: structured, self‐guided modules foster independent skill practice (e.g., stimulus control, worry time, problem‐solving), whilst therapist input reinforces and personalizes behaviour change strategies (Mohr et al., 2013). This dynamic interplay may be particularly effective in enhancing uptake of techniques such as behavioural activation and cognitive restructuring, which, in turn, may further promote sleep and wellbeing. These insights warrant further investigation to inform optimization of hybrid CBT interventions targeting comorbid sleep and emotional difficulties.

Of importance to employers and employees, this qualitative process evaluation identified that having a private space to take therapy session calls was vitally important. For this reason, it is likely that working from home during COVID‐19 improved engagement with the programme, providing greater control over the therapy environment. Those who were interviewed tended to prefer keeping their mental health concerns out of the workplace. It was discussed within the qualitative research team that this may be due to the nature of working relationships within different work settings, representing a preference by employees, as opposed to any direct stigma received from colleagues (no accounts of direct stigmatization were apparent within interview data). Inevitably, specific work context and openness of conversations about mental health vary per organization. The opportunity to engage with therapy away from workplace surveillance, whether real or perceived, may have fostered openness, reduced internalized stigma, and allowed for deeper reflection (Carolan & de Visser, 2018). This aligns with broader theories of mental health help‐seeking in occupational contexts, where stigma and perceived risk to professional identity often deter help‐seeking and engagement (Cameron, 2011; Tsantila et al., 2023). This is a broadly applicable contextual insight for developers of digital sleep and mental health interventions in the workplace. Indeed, if SLEEP were to be trialled in a different context, particularly with employees who conduct their work in open‐plan office settings, the aspect of privacy would have to be considered thoroughly. Engaging with stakeholders prior to intervention development (e.g., via Patient Public Involvement) may help to determine which intervention delivery modalities are appropriate for different workplace settings.

Interventions like SLEEP will also need to account for organizational cultures that may undervalue sleep or view sleep difficulties as a personal failing rather than a legitimate health concern. Cultural norms around productivity, availability, and emotional stoicism may create subtle barriers to uptake, especially in settings or industries with rigid hierarchies or limited wellbeing infrastructure. These findings suggest that future implementation should be accompanied by workplace‐wide efforts to normalize conversations around sleep and mental health, reduce stigma, and create enabling environments.

### Limitations

Participant evaluations of the intervention were overwhelmingly positive, although one negative experience was recorded. Whilst participants were selected at random to be contacted to participate in an interview from within each arm of the main trial, selection bias may still have impacted findings. Participants who had a negative experience or experienced little to no change may have chosen not to respond to the invite. The research team ensured and made clear to participants that they would not be interviewed by any of the researchers who had been allocated as their therapist. Nonetheless, it is possible that some participants may have avoided giving negative feedback, particularly via the therapist team who were also involved in the evaluation.

Interviews conducted as part of this evaluation were relatively short (mean length: 27 min), which may have limited the depth of data available. There are several reasons this may be. The nature of this study being with working people and conducted within working hours likely played a role in participants giving short, pragmatic responses. Several members of the research team had limited qualitative experience; therefore, a structured interview guide was used, as opposed to semi‐structured. This may have led to reduced rapport due to the rigidity of the topic guide. Prompts may not have been frequently used, which may have limited the length and depth of responses. The majority of participants had good experiences and were highly engaged; therefore, asking questions about barriers to engagement may simply have yielded short responses.

Individual‐level tailoring was essential to the success of the SLEEP intervention; however, an important limitation of this study is that therapist sessions were not recorded to assess fidelity. Information about exactly how each individual's sessions were tailored was not collected, particularly whether they focused more on CBT‐I versus ER components of the programme; hence, it is difficult to interpret precisely which components impacted which outcomes. This would have been a useful part of the process evaluation to help understand the direction of effects as to whether better sleep alone impacted improved mood, or whether tailoring towards the ER component of the intervention improved mood either alongside or as a result of improvements in sleep.

With regards to the generalizability of the findings, given the predominance of office‐based and hybrid workers in our sample, and the COVID‐19 context, the findings may not be readily transferable to frontline roles such as healthcare, construction, or transportation, where work patterns are typically more rigid and less amenable to remote engagement. Future research should explore the feasibility and acceptability of similar interventions in these settings, taking into account the structural and scheduling challenges faced by these workers.

## CONCLUSIONS

Participant experiences of SLEEP, a hybrid dCBT‐I + ER intervention, were positive overall, with improvements to sleep and wellbeing exceeding expectations of several participants who were interviewed. Triangulation of quantitative and qualitative findings was harmonious, where improvements to sleep and wellbeing were substantiated by quantitative data from the main trial. Regular therapist contact helped to facilitate behaviour change throughout the digital programme, wherein it is proposed that tailored problem‐solving increased engagement. Indeed, therapist contact was vital to the success of SLEEP. As this trial was conducted during COVID‐19, in the ‘working from home’ period, this context is likely to have enhanced intervention engagement. Lastly, findings highlighted that private spaces were important in enabling successful online therapy delivery; this represents an important insight for intervention developers and employers looking to implement a programme such as SLEEP in a workplace environment.

## AUTHOR CONTRIBUTIONS

**Anna Hurley‐Wallace:** Conceptualization; investigation; writing – original draft; methodology; validation; visualization; writing – review and editing; formal analysis; data curation; software; project administration. **Sophie Fletcher:** Investigation; writing – original draft; formal analysis; data curation; writing – review and editing; visualization; project administration; conceptualization; validation. **Talar R. Moukhtarian:** Investigation; funding acquisition; writing – original draft; writing – review and editing; formal analysis; supervision; validation; conceptualization; resources; project administration. **Krishane Patel:** Conceptualization; investigation; funding acquisition; writing – original draft; writing – review and editing; visualization; supervision; formal analysis; software; methodology. **Agatha Payne:** Investigation; formal analysis; writing – review and editing. **Tabitha Jackson:** Investigation; formal analysis; writing – review and editing. **Carla Toro:** Conceptualization; funding acquisition; writing – review and editing; supervision; validation. **Guy Daly:** Conceptualization; funding acquisition; writing – review and editing. **Sean Russel:** Conceptualization; funding acquisition; writing – review and editing. **Lukasz Walasek:** Conceptualization; writing – review and editing; supervision; writing – original draft. **Nicole K. Y. Tang:** Conceptualization; investigation; funding acquisition; writing – original draft; writing – review and editing; visualization; validation; methodology; formal analysis; supervision; resources. **Caroline Meyer:** Funding acquisition; writing – review and editing; supervision; conceptualization; validation.

## Supporting information


Data S1.

Data S2.

## Data Availability

The data that support the findings of this study are available from the corresponding author upon reasonable request.
