# Supplementary material for: ‘Better sleep, better wellbeing’: Qualitative process evaluation of a hybrid, digital cognitive behavioural therapy programme for employees with sleep and emotion regulation problems
Source: Br J Health Psychol. 2025 Dec 4;31(1):e70041. doi: 10.1111/bjhp.70041 (PMC12676197; doi:10.1111/bjhp.70041)
Supplement: Supplementary file 1 — Data S1. [file BJHP-31-0-s001.docx]

**EMPLOYEE INTERVIEW QUESTIONS – INWORK**

This interview with the employees relates to the SLEEP and REST trials.

The interview will be recorded and run-on MS Teams online video call which will take around 45 minutes.

**Introduction**

This interview will ask you questions about your thoughts on the (SLEEP/REST) programme, including your overall experience of the programme, how it has impacted you, any barriers to taking part or anything you would have liked to be different.

Before we start the interview, can you please confirm which trial you took part in as a participant [tick as appropriate]?

• REST ☐

• SLEEP ☐

To help me capture all the important information, this interview will be audio recorded. Everything that we discuss will be confidential and your recording will not be shared with anyone outside the research team. We will not be reporting on individual responses, and no one will know how you have personally responded.

Do you have any questions before we begin? **[allow time for them to think and respond]**

If anything crops up during or after the interview that you would like to discuss, or that you have a question about, please do feel free to email the research team at wmg-mhpp@warwick.ac.uk.

We will start the session with going over your consent form again and then move on to the interview questions.

**[Share screen with their consent form]**

If you are ready, I will start the recording now **[start recording]**.”

**[Complete consent- Read out the statements again and ask them to confirm they understand/say yes; add your name and date; save “read only” as another copy with your initials]**

**Prompts** (can be used throughout)

- Can you tell me more about that?
- Can you expand on that?
- What do you mean by [repeat word/ statement]?

**Questions (to be asked in numbered order)**

1. What was your overall experience of being in the programme?
   - What aspect of the programme did you like the most? (e.g. educational components, practical solutions/tools, therapist contact, online/digitalisation, flexibility around commitments)
   - Why did you like [*repeat back answer*]?
   - What aspect of the programme did you like the least? (same prompts as above)
   - Why didn’t you like [*repeat back answer*]?
2. What did you think about the format in which the programme was delivered? (e.g. length, resources, content)

- How was your experience with the digital platform/online content? (e.g. usability/ user-friendly/ easy to use?)
- [SLEEP ONLY] How was your experience with your therapist(s)?^[[1]](#endnote-1)^
- [SLEEP ONLY] What did you think about the topics covered during the online therapy sessions?
- [SLEEP ONLY] Did you feel there were a suitable number of therapy sessions?
- What do you think about how long the programme lasted? (too long/ too short/ the right length)
- What do you think about the online/digital nature of the programme?

1. How did you feel that the programme was tailored to your needs? (general prompts – e.g. can you tell me more about that?)
2. What were your initial expectations of the programme, if any?
   - Did the programme meet your expectations?
   - What impact did you anticipate the programme would have on your sleep, and did it meet those expectations?
   - Can you tell me about any changes to your sleep that you have experienced?
3. What impact did you anticipate the programme would have on your physical or mental wellbeing, and did it meet those expectations?

- Can you tell me about any changes to your physical or mental wellbeing that you have experienced?
  - As a result of the programme, did you experience any changes in other areas of your life? (e.g. social, work-life balance)

1. Were there any barriers that you experienced during your participation in the programme?
   - Were you able to complete the required activities around your existing commitments? (e.g. attend appointments, complete online content)
   - Did you have access to a private space and/or necessary IT equipment?
2. Did you have any concerns about stigma during your participation in this study? (e.g. from employer, colleagues, family)

- [If so, can you tell me more about this?]

1. If you had the opportunity, what would you most like to change about the programme?
   - Is there anything that you wish had been in the programme, but was not there?

**CLOSE**

“Thank you for taking the time to give us your feedback on the (SLEEP/REST) study. I will now stop the recording **[stop recording].**”

1. Note that some participants in SLEEP had 2 or 3 different therapists. [↑](#endnote-ref-1)
