# Supplementary material for: ‘Better sleep, better wellbeing’: Qualitative process evaluation of a hybrid, digital cognitive behavioural therapy programme for employees with sleep and emotion regulation problems
Source: Br J Health Psychol. 2025 Dec 4;31(1):e70041. doi: 10.1111/bjhp.70041 (PMC12676197; doi:10.1111/bjhp.70041)
Supplement: Supplementary file 2 — Data S2. [file BJHP-31-0-s002.docx]

Codebook – SLEEP interviews 2022

| Code name | Description | Files | References |
| --- | --- | --- | --- |
| **Additional programme suggestions** [FRAMEWORK CODE/ TOP-LEVEL] | Suggestions for specific content areas to be added to platform or program. | 0 | 0 |
| Suggestion - better or broader advertisement | Says if better advertised the programme could have helped colleagues. Did mention however that people also need to want to change. | 3 | 5 |
| Suggestion - better signposting mechanisms | Better signposting mechanisms would be helpful for those completing the programme who still require further support and for those where the programme doesn't fully meet their needs | 1 | 3 |
| Suggestion - content - dreaming | Suggestion to include content within the programme to aid understanding of sleep and dreaming functions. | 1 | 3 |
| Suggestion - content - sleep medication | Suggestion to include content on sleep medications, similarly e.g., to other health-information websites. | 1 | 2 |
| Suggestion - content - trauma | Suggestion to include more psychoeducational content/techniques on past traumas that impact and are triggers for certain sleep issues. Noted may not be applicable/relevant for everyone | 1 | 2 |
| Suggestion - early intervention for young people | Talks about making content available to younger people, students, children etc , who could benefit longer-term. | 2 | 2 |
| Suggestion - focus group sessions | Talks about potentiality for focus group sessions to feedback on each others sleep progress. | 1 | 2 |
| Suggestion - initial welcome call or intro | To make introduction to programme more streamlined and to reduce the 'to and froing', suggestion to have an introductory welcome call/ more info to explain the programme and how it will work. | 2 | 4 |
| Suggestion - integrated alert or reminder to complete required activities | Would be useful to have some sort of integrated reminders of what the participant has to do (e.g. diary, complete survey, access to new content etc), however noting that they did receive an email reminder from the team which was helpful | 1 | 1 |
| Suggestion - lead-on CBT sessions | Suggestion that having CBT-based sessions leading on from the sleep therapy components would be nice. Follow-on sessions/ additional. | 3 | 5 |
| Suggestion - more for employees with children | Suggests there could be more specific content for employees with young children. | 1 | 1 |
| Suggestion - more scientific sleep recording | Discusses potential for more advanced sleep recording and that this would be of interest to them as a participant. | 1 | 2 |
| Suggestion - paper version of online content |  | 1 | 1 |
| Suggestion - showcase success stories | Showcase real testimonials as a way of encouragement and engagement to others. | 1 | 2 |
| Suggestion - WhatsApp communication function | Suggests WhatsApp function for communication (as opposed to email) | 1 | 3 |
| **Barriers to engagement** [FRAMEWORK CODE/ TOP-LEVEL] | Barriers to engagement and subsequent behaviour change. | 0 | 0 |
| Advice on work-life balance - not applicable | Talks about content on work-life balance not being applicable. Linked with other code 'suggestion to focus on the root of the problem' i.e. dealing with work pressure/ stress itself. | 1 | 3 |
| An insomnia 'imposter' | Refers to feeling like an 'imposter' in the study or like they shouldn't be there because others have greater need. | 1 | 2 |
| Avoiding bringing mental health to work | Avoidance/ reluctance to discuss mental health issues/ struggles with colleagues and managers. This is related to internalised beliefs about mental health stigmatization, rather than because of any stigmatization from colleagues directly. | 7 | 17 |
| Busy schedule | Talks about busy schedule being a barrier to completing modules and making subsequent changes. | 6 | 25 |
| Comparison to previous therapy | Compares programme to previous sleep therapy which included SRT. | 1 | 6 |
| Content too COVID-19 focused | Focus of the trial was too much on COVID and ignoring other triggers, issues and problems | 1 | 1 |
| Diary - challenging to record accurately | References to the sleep diary being challenging to record accurately. This is not necessarily described as a 'barrier', more as a challenge in that it was difficult to remember and state specific e.g. times (minutes) of waking during the night. | 5 | 14 |
| Diary - repetitive and boring | Boring repetition of filling in diary, having to remind self to complete task, Understood the need to do this, but boring nonetheless. | 2 | 3 |
| Diary-tracker discrepancy | Expresses concern about how accurately they are able to fill in the sleep diary, verses what is shown on the sleep tracker. Worry that this will impact the study results often mentioned. | 3 | 5 |
| Diminished responsibility | Reduced responsibility on behalf of participant. Awareness of the underlying issue, but not executing despite knowing the motivation. | 1 | 4 |
| Employers should be more proactive | Talks about the need for employers to be more proactive in wellbeing strategies for staff. Relates to 'focus on the root of the problem' code | 1 | 4 |
| Exercise - lack of motivation | Talks about wanting to change exercise behaviours and discussions of this with therapist, but struggles with initial motivation to change or to begin forming any new habits. | 1 | 5 |
| Familiar with diet and exercise content | Talks about not engaging with diet/ exercise content as already known info. | 1 | 1 |
| Familiar with relaxation techniques | Familiar/ already practices mindfulness, meditation, relaxation, breathing. Also code yoga references if used for relaxation purposes. | 2 | 4 |
| Familiar with sleep restriction | Talks about having tried sleep restriction before. Links to chronic insomnia. | 1 | 3 |
| Familiar with stress-management | Talks about having prior knowledge of stress-management techniques including time-management and worry management. | 1 | 3 |
| Felt like a data point | Participant says they felt like a data point - that they were only there to give data. | 1 | 1 |
| Guilty about taking time from work | Talks about feeling like they should be able to complete programme during work time, but sense of guilt that this is taking away from their focused work time. Sees programme as benefitting themselves, not work/ employer. | 2 | 3 |
| I expected more from 'CBT' | Talks about expecting more from CBT. Has a prior understanding of this and expects depth and exploration of 'why'. | 1 | 8 |
| Initial scepticism of programme | Initial negative preconceptions and concerns about programme, sceptical about how beneficial the programme would be for them (e.g. sceptical about how helpful online content would be). | 1 | 2 |
| Initial worry about impact of sleep restriction | Expresses initial concern/ worry at the beginning of the programme about how SRT (SRT = Sleep Restriction Therapy) will increase tiredness and impact concentration e.g. ability to complete work, or drive. | 1 | 2 |
| Internalised stigma or beliefs - mental health | Stigmatic attitudes/beliefs of the participant themselves. Taboo mental health/ reluctance to broach topic. For example, references to mental health issues negatively impacting others' opinions towards them. Code direct stigmatization by others separately. | 4 | 12 |
| Interruption from work | Talks about being interrupted when taking therapy session calls at work. | 1 | 2 |
| Issues finding a private, confidential space | Issues/ difficulties accessing a private space to take therapy calls. This may be at work (e.g. an open office) or at home (e.g. partner working from home). Relates to issue of confidentiality when engaging in therapy content or calls in a work environment. | 2 | 11 |
| IT literacy | IT or computer literacy/ competency of individuals noted as a potential barrier to using the online platform. | 3 | 3 |
| 'It's just in your head' | Perception that programme focuses more on hypothetical worries and how to manage them, less focus on how to deal with stressors that objectively happen (i.e. facing pressure at work). Links to perception of ignoring root cause of sleep or emotional problems (coded under 'Suggestion for more focus on the root of the problem'). | 1 | 4 |
| 'It's not going to change what happened' | Statement that although it is nice 'having someone to listen' (linked code), the context or negative situation that happened cannot be changed. Stated as a reason for not previously engaging with therapy services. | 1 | 2 |
| Juggling priorities - lifestyle vs sleep | Talks about having to manage priorities when managing sleep verses other aspects of lifestyle e.g. socialising, busy schedule etc. | 2 | 4 |
| Limited IT equipment during work day | Raises issue of having limited IT equipment during the work day, due to the type of work that they do. This made it hard to facilitate therapy contact sessions during working hours. | 1 | 2 |
| Limited therapy time | Talks about limited therapy time leading to disengagement with therapy or 'holding back'. May link to rapport development - 'therapeutic alliance'. | 1 | 5 |
| Misunderstanding of the study purpose | Misperception that study was just measuring/assessing sleep as is currently, rather than delivering an intervention to promote change/improvement | 3 | 5 |
| More focus on the root of the problem | Suggests that the programme could better address causal factors/ root of the sleep or emotional problem i.e. dealing with pressure at work, going to line manager, dealing with other people. Rather than focusing just on what the individual can control (as is the case in CBT/ ACT therapies). Links to code 'it's just in your head'. | 3 | 5 |
| Negotiating with sleep partner | Explains having to explain the study with sleep partner and negotiate with them. | 4 | 5 |
| Online content - too wordy | Found the chunks of texts a bit long. | 1 | 1 |
| Online content - unable to skip ahead | Talks about wanting to be able to skip ahead in online content, to do bigger chunks at a time. | 1 | 1 |
| Online nature | Online nature of content doesn't suit learning style. Prefers to have paper, doesn't like being on a screen. Also relates to time spent to log on to website when have a busy schedule, but coded this separately as 'busy schedule' | 1 | 6 |
| Opened a can of worms | Participant had begun to manage/deal with problem during the course of the programme but ran out of time to finish it. Left to finish dealing with problem on their own. Links to additional signposting and content suggestions. | 1 | 6 |
| Order of content and sessions | Therapist sessions and online programme didn't quite align - would have preferred to study the content directly before therapy session as it was hard to remember what I had studied from the previous week | 2 | 9 |
| Preference to have same therapist throughout | Talks about experience of having a therapy session covered by a different therapist and preference to have the same person throughout to streamline the programme. Includes not having to repeat oneself, not having to get to know two different therapists etc. | 4 | 5 |
| Programme comprehension | Talks about worries relating to programme comprehension/ understanding content and/or advice given. Code any references to worries about understanding/ comprehension, regardless of cause (e.g. language barrier, disability, education level). | 2 | 5 |
| Programme length - too long | States length of programme was too long for their needs and/or liking. | 2 | 4 |
| Programme length - too short | Duration of the programme too short. Could have benefitted/got more out of the programme if it was longer | 1 | 3 |
| Psychoeducation - didn't match my situation | States that online psychoeducation did not match their situation, and this left them feeling defeated at the onset of the study. | 4 | 6 |
| Sessions could have been more flexible | Talks about session could have been more flexible i.e. some deviation outside of study time-periods allowed. | 1 | 1 |
| Sleep content was broad | Talks about sleep content being quite broad, not everything being applicable, but some aspects being helpful. Neutral statement. | 5 | 7 |
| Sleep inertia (morning fatigue or tiredness) | Talks about sleep inertia (morning fatigue or tiredness) impacting their sleep quality scores, and realising that this was a barrier to recorded improvement. | 1 | 1 |
| Sleep problems are mild | Talks about sleep problems being mild/ not insomnia/ sub-clinical. | 4 | 9 |
| Sleep restriction - incompatible with shift work | Talks about not following sleep restriction strictly as this did not work well with early shift work. Note that shift work should have been screened out of study. | 2 | 3 |
| Sleep restriction not relevant | Talks about sleep restriction not being relevant for them, despite it being a major component of the intervention. | 1 | 1 |
| Sleep restriction was the hardest | Talks about sleep restriction being the most difficult/ challenging aspect of the programme. | 4 | 13 |
| Technical issues | Talks about some technical issues experienced with online modalities. | 4 | 4 |
| Tedious sign-up process | Initial sign-up, consenting etc. not very smooth or streamlined, a lot of 'to and fro'. | 1 | 2 |
| Therapist - critiqued competency | Critiques therapist competency. States they were out of their depth, didn't know what they were doing. Variety of quotes available. | 1 | 9 |
| Therapy session content - boring | States that therapy session was boring and they zoned out. | 2 | 2 |
| Therapy sessions - lacked depth | Talks about therapy sessions lacking depth. Annotation here that they were asked to do worry time but did not do. | 1 | 13 |
| Time-commitment | Talks about programme being a significant time-commitment. Negative framing. | 4 | 12 |
| Tracker - annoying or irritating | Reference to tracker watch as annoying or irritating. Include skin irritation references. Include finding it generally annoying to wear/ uncomfortable. | 4 | 7 |
| Tracker - data privacy concerns | Talks about concerns regarding the tracker watch and the data it is collecting. Concerns about it recording everything they are doing. | 1 | 5 |
| Tracker - not aesthetically pleasing | Talks about tracker not being nice looking/ vanity comment. | 1 | 2 |
| Tracker - poor quality (perceived) | Thought it was cheap and uncomfortable, difficult to use compared to commercially available trackers e.g. FitBit. Felt we could have got better quality ones. Consensus from other trial participant. | 1 | 3 |
| Tracker - too much at once | Talks about being busy with work and social life, and finding the tracker, diary and online content were a lot to do at once. | 1 | 1 |
| Transcripts should be included with meditation audio | Suggestion to include audio transcripts for all materials, rather than just some. Found this helpful for the explanatory videos, but no transcript available for audio meditation. | 1 | 2 |
| Unclear communication | Not clear communication regarding the requirements of the participants to take part in the trial (e.g. when needed to be at home, what dates they were taking part). Include unclear logistics communication. | 4 | 9 |
| Worried about diary inconsistencies | Noted worry about feeling in diary inconsistently (throughout programme). Code 'diary-tracker discrepancy' references separately. | 1 | 2 |
| Would have liked in-person | References to desire for in-person/ face-to-face therapist contact sessions, as opposed to or as well as online. | 2 | 3 |
| **Behaviour Change Taxonomy (BCTs)** [FRAMEWORK CODE/ TOP-LEVEL] | Codes that refer to behaviour change taxonomy specifically. | 0 | 0 |
| Sleep - behaviour substitution 8.2 | Specific references to substituting one behaviour with another to improve sleep. For example, replacing screen time with a sudoku. BCT - behaviour substitution 8.2. If occurs regularly, also code habit reversal 8.4. | 3 | 8 |
| Sleep - behavioural experiments 4.4 | Talks about testing-out or trialling the new sleep schedule, trying out the evidence-based advice to see what the result or outcome is. BCT - behavioural experiments 4.4. | 5 | 12 |
| Sleep - behavioural practice 8.1 | References to practicing a persevering with a new sleep schedule or routine. Includes talk about not always being successful. Relates to habit formation. Only code as habit formation if there is specific refence to the context eliciting the sleep behaviour i.e. waking up prompts getting up, getting bag/ coffee. BCT 8.1. | 19 | 48 |
| Sleep - credible source 9.1 | References to the online content by experts in the area and how this was reassuring or valid/good information. BCT 9.1. | 5 | 8 |
| Sleep - feedback on behaviour 2.2 | References to monitoring of behaviour (with feedback integral in programme), helped them to make changes to their sleep behaviours. Include references to reporting back to therapist and discussing the weeks progress. BCT 2.2. | 12 | 27 |
| Sleep - habit formation 8.3 | Code overlaps with 'sleep behaviour change - behavioural practice.' Only code as habit formation if clear association is built between the context and the new behaviour. For example, waking up prompts routine of get bag, shower etc. BCT 8.3. | 3 | 3 |
| Sleep - information about antecedents (predictors) 4.2 | Providing information about social and environmental situations or events that reliably predict performance of behaviour. BCT 4.2. e.g. might refer to circadian rhythm, presence of light stimulus predicting wakefulness OR refers to mood scores impacting sleep scores. | 7 | 19 |
| Sleep - problem solving 1.2 (therapist tailoring) | Refers to therapists prompting the person to analyse factors influencing behaviour/ review the weeks progress, and discuss strategies for how to improve (through tailoring) e.g. removing a barrier or adding an incentive. BCT 1.2. | 17 | 48 |
| Sleep - reduce negative emotions 11.2 | References to making time for emotion regulation, or building emotional regulation skills, specifically to facilitate the improvements to sleep behaviours. For example references to I've already done the thinking, which helps me sleep' or 'I am now able to relax, which helps me nod off'. BCT 11.2. | 12 | 25 |
| Sleep - restructuring the physical environment 12.1 | For example adding blackout curtains. Relates to Enablement. | 2 | 4 |
| Sleep - self-monitoring of behaviour 2.3 | Talks about building self-discipline, being more disciplined, sticking with the 'rules' of the programme. Reflects self-monitoring element of programme BCT 2.3. | 8 | 18 |
| Sleep - self-monitoring of outcomes of behaviour 2.4 | Self-monitoring of outcomes of behaviour. An example of this in this particular trial is reducing napping resulting in more energy/ time - positive outcomes. 2.4 | 6 | 12 |
| Sleep - suggestion - increase feedback via tracker 2.2 | Suggestion for increased feedback on behaviour by utilising tracker within intervention programme. BCT 2.2. AHW notes: check this code against 'curious about tracker results' - next iteration. | 3 | 3 |
| Sleep - suggestion - increase self-monitoring of behaviour 2.3 | Talks about online content not giving them the opportunity/reminder to be accountable/check up on what they're doing, Suggestion that purposeful self-monitoring of sleep behaviours could be added into the online programme. BCT 2.3. Note that therapist/therapy session helps them to be accountable through feedback on behaviour (BCT 2.2). | 1 | 1 |
| Time-management - action planning 1.4 | Found the content and resources on prioritising (i.e. prioritising matrix) helpful. Developing a new skill. BCT 1.4. | 4 | 7 |
| **Context** [FRAMEWORK CODE/ TOP-LEVEL] | Contextual factors impacting outcomes/ behaviour change. | 0 | 0 |
| Accessed other therapy during trial | Talks about accessing other therapy service during trial, to tackle mental health issues specifically. Relates to code 'sleep as an access point'. | 1 | 3 |
| 'Better version of me, working from home' COVID-19 | Suggests they are a better version of themselves working from home - working from home suits their personality/ lifestyle. Positive. | 1 | 2 |
| Chronic insomnia | Description of chronic insomnia over several years. | 2 | 6 |
| Cognitive distortions (negative thoughts) | Talk about negative thoughts or cognitions/ cognitive distortions they have experienced. Relates to co-morbid low mood/depression symptoms. Descriptive. | 1 | 2 |
| Comparison to sleep partner | Direct comparison of participants sleep to sleep partner. | 1 | 1 |
| COVID-19 not an agile working environment | Talks about COVID-19, online working, not being an agile working environment, creating work stress within the team, etc. | 1 | 3 |
| COVID-19 stunted improvements to social life | Reflection that COVID-19 pandemic (social distancing etc) potentially stunted improvements to social life / work-life balance, that could have been elicited by the study in a different circumstance. | 4 | 4 |
| Environmental or external factors impacting trial | Circumstances/situations that would make normal routine different and could impact on sleep or confound intervention (e.g. daylight hours in summer, different routine being on holiday). See linked memo. | 12 | 29 |
| Expectations - generally positive | Going into programme thinking that it would be helpful, useful, would make some improvement to their current issues/problems | 7 | 10 |
| Expectations - 'life changing' | They expected that the programme would change their life. Unrealistic. | 1 | 1 |
| Expectations - tips for improvement, not cure | Expected some tips for improving sleep that they could apply. Understood that there is no magic cure or quick fix. | 7 | 13 |
| History of clinical sleep problems | Description of assessments or diagnoses of clinical sleep problems including sleep apnoea, parasomnias, restless leg etc. Include references to hospital and specialist investigations of sleep problems. Do not include prescription of sleep medications by GP - code separately. | 1 | 1 |
| I don't want to burnout | Talks about experience of burnout at work. | 1 | 3 |
| I don't want to worry other people with my mental health | Talks about not wanting to put their emotions on others, or worry their family/ friends/ colleagues with their own mental health problems. Would rather deal with them on their own. | 1 | 3 |
| 'I was desperate to try anything' | In vivo code - this is a contextual facilitator. Desperation to try anything clear from client context. Relates to long-term insomnia. | 3 | 3 |
| Improved sleep due to restrictions COVID-19 | Improvements to sleep relating to COVID-19 restrictions i.e. Not having to commute, better work/life balance, rather than related to the trial. | 1 | 1 |
| Likes having the choice to be sociable or not | Talks about the importance of choice to socialise verses not. Preference for having this choice and control over social engagement/ interpersonal interactions. | 1 | 2 |
| Low mood - description of symptoms | Description of classic symptoms of low mood/ depression. Indicates potential clinical comorbidity with sleep problem. | 2 | 3 |
| Mental health - better when I'm busy | References to mental health issues feeling less prominent/ easier to manage when distracted or busy with tasks. | 1 | 6 |
| Mental health deterioration leading up to study | Description of deteriorating mental health in the months/ years leading up to participation in the study. | 2 | 4 |
| My depression might affect other people | References to low mood/ depression impacting other people to make them feel low mood/ depressed. Or wishing they hadn't engaged in conversation in the first place. | 1 | 3 |
| My manager was surprised, but supportive | Neutral statement about manager being surprised, as well as supportive, of participation. | 1 | 1 |
| No concerns about stigma | Participant states that they did not have any concerns about stigma related to study participation. | 11 | 15 |
| No initial expectations | Had no initial expectations of the programme/ no expectations to compare to. | 10 | 25 |
| Our lives are online COVID-19 | Talks about 'we are online anyway/ working from home anyway' - contextual factor which also contributes to study uptake. | 10 | 15 |
| Pre-existing medical conditions | Describes pre-existing medical conditions that impact levels of fatigue. | 1 | 2 |
| Prior interest in sleep and psychology | Took part due to general interest in sleep and psychology, rather than due to a major sleep problem per se. | 5 | 10 |
| Returning to hybrid working COVID-19 | Talks about a struggle returning to hybrid work, particularly on office days, after the trial has ended. Relates to sleep schedule management long-term. Change in working pattern affecting sleep pattern. | 2 | 4 |
| Social context - description | Description of social context, including their living circumstances, their interpersonal relationships. Include talk about changes in context over time. | 8 | 24 |
| Stigmatic attitudes of others | Stigmatic beliefs of family, friends, colleagues, managers about sleep/mental health generally, the programme etc. Relating to the others stigmatic attitudes, rather than the participants perception/internalisation | 2 | 6 |
| Work context - description | Description of the working context/ work environment. Includes descriptions of type of work and time/ hours. Code references to interpersonal 'working relationships' separately, | 9 | 10 |
| Working from home creates a private space COVID-19 | Discusses that working from home during the pandemic enables them to access the resources and therapy sessions in a private space. | 6 | 9 |
| **Facilitators to engagement** [FRAMEWORK CODE/ TOP-LEVEL] | Facilitators to engagement and subsequent behaviour change. | 0 | 0 |
| A 'holistic' approach | Positive about programme, talks about the programme being 'holistic', well-rounded, addressing other factors relating to sleep etc. Relates to health psychology - biopsychosocial approach. | 4 | 6 |
| Ability to access resources afterwards | Ability to access resources long-term was viewed as positive. Allows flexibility for completion. | 3 | 4 |
| Benefitting the research | Refers to being present and actively engaged with the study because of the potential benefit of participation to the research (i.e. Others who need it will benefit from my engagement). | 1 | 5 |
| Diary - useful | Found the sleep diary insightful/useful. | 4 | 11 |
| 'Digital alone would never, never work' | Felt the face-to-face element was important and that online-only would not have worked for this type of programme. | 2 | 3 |
| Downloadable worksheets - useful | Talks about downloadable worksheets being useful - so they didn't always have to go online. Liked the ability to print information. | 3 | 5 |
| Easy-to-access resources | Statement that resources were easily accessible (being online). | 10 | 18 |
| Eating well - helpful advice | Talks about eating advice being helpful - not too specific and not too generic - correct level of detail. | 1 | 2 |
| Employer was supportive | Talks about employer being supportive or showing support for their participation in the programme, including using work resources to engage with programme. | 5 | 9 |
| Flexibility of online content | Ability to be able to pick up and put down/complete online content at a time convenient for them. E.g. able to complete content in quieter period around work | 5 | 12 |
| Flexibility of sessions | Found it helpful that sessions could be moved if there was a diary clash, and also found flexibility of the content of the sessions helpful - i.e. if something wasn't relevant then they could focus on something else | 10 | 15 |
| Going beyond generic advice | More than just normal, 'standard' or 'generic' advice. Providing detail/information beyond what is expected. Positive. | 5 | 7 |
| Had access to necessary IT equipment | Participant talks about having adequate access to IT equipment needed for trial. | 12 | 14 |
| Harmony of online content and therapy content | Talk about the benefits of the online content and therapist sessions working in harmony/ synchronously together. For example, include references to the therapy sessions offering an opportunity to tailor particular teachings from the online content, or a chance to reflect and apply knowledge. | 12 | 21 |
| Having someone to listen | Talks about the value of having someone to listen and take on board their feelings and emotions, before providing suggestions. This code relates to motivational interviewing skills that are present in therapy sessions and potentially in interviews as well (active listening). | 5 | 7 |
| I found time to complete everything | States that they had enough time to be able to complete all the online material and sleep diary etc. | 11 | 16 |
| I learnt something new | Talks about there being lots of new information and learning new techniques and skills. | 10 | 17 |
| Importance of therapist position as 'outsider' | Talk about the positive experience of therapy with someone 'outside of the situation', a 'stranger', someone 'independent', and other synonyms. | 1 | 4 |
| Interactive online content - engaging | Talks about interactive activities in online programme being engaging/ less boring. | 9 | 13 |
| Multi-media formats - engaging | Talks about the use of a variety of media/ audio-visual i.e., videos, text, audio, links on the online platform being engaging/ appealing. | 10 | 17 |
| My partner was supportive | Talks about partner being fully onboard and supportive of participation. | 1 | 1 |
| Online content - time estimates useful | Found the provision of time estimates for each section of the online content useful to be able to manage/allocate time accordingly. | 1 | 2 |
| Opportunity to change | Viewed the programme as an opportunity change. Overlaps with code for chronic insomnia. Chronicity = seeking intervention. | 1 | 1 |
| Programme length - good | Both length of programme and number of sessions was about right. | 16 | 27 |
| Progressive learning | Talks specifically about the progression or layering of topics and how they gradually increased learning/ understanding. Gradually increasing complexity of topics, positive. | 2 | 3 |
| Provision of video transcription | Provision of transcriptions reduced the time it took to get through programme - facilitator. | 1 | 1 |
| Psychoeducation - normalising sleep patterns | Talks about how the normalisation of different sleep patterns and individual variation in sleep was helpful to realise. Reassuring that their normal may be different to others'. This was present in the psychoeducation part of the online content, and reiterated in therapist sessions. | 7 | 14 |
| Psychoeducation - relevant and applicable | Found the psychoeducation/ information relevant and easy to apply to their own lives. Some talk about the information helping participants understand what has been fuelling their sleep problems. | 18 | 37 |
| Psychoeducation - science behind sleep restriction | Found learning the science behind sleep restriction encouraged them to engage with this element of the programme (more so than therapist encouragement). | 1 | 1 |
| Reiterated what I already knew | Talks about knowing about how to address sleep problems/ manage emotions, but programme reiterated information and helped to bridge the intention-behaviour gap. | 8 | 14 |
| Releasing content week-by-week | Discusses the benefits of releasing content weekly, as opposed to reading ahead. | 4 | 8 |
| 'Small chunks' of content | Talks about small chunks of content making the programme more engaging. Content was digestible using a series of shorter interactive activities. | 2 | 2 |
| Smooth logistics | References to logistics being smooth e.g. DHL delivery easy to move. | 6 | 6 |
| Suitable amount of online content | Felt that there was a good amount of online content/it was long enough. Specifically relating to online content, not programme as a whole/inclusion of therapy sessions | 2 | 3 |
| Suitable number of therapy sessions | If there were a suitable number of therapy sessions (related code identifying change as a result of intervention, but this relates more to overall timeframe, see code definition) | 12 | 15 |
| Teams was as good as face-to-face | Talks about MS Teams videoconferencing being just as good/ the same as face-to-face. No additional benefit of in-person therapy sessions. | 2 | 2 |
| The team were lovely | References to interactions with the wider study team being positive. E.g. people were lovely. | 3 | 4 |
| Therapeutic alliance | Reflections on therapist session that refer to working together, collaboratively deciding on what to do, description of the therapist as an 'ally'. | 15 | 28 |
| Therapist - competent | Reference to therapist being competent - skilled and knowledgeable, offering valid advice. This code refers to role competence as a SLEEP therapist i.e. competency within the remit of the trial. | 14 | 20 |
| Therapist - warmth | Talk about feeling comfortable with the therapist, due to them being 'understanding', warm-natured, non-judgemental, empathetic, etc. | 11 | 17 |
| Therapist aids accountability | Talks about being accountable or just doing it because of someone monitoring. Links with BCT code - feedback on behaviour 2.2. | 6 | 11 |
| Therapist providing clarification | Opportunity to clarify questions or ask the therapist to provide further explanation on specific topics was helpful. | 13 | 26 |
| Therapy sessions 'the best part' | Statement that therapist sessions were 'the best part' of their experience. Facilitator relating to therapist competency and rapport, and ability to problem-solve/ tailor. | 7 | 11 |
| Therapy sessions well spread | States that therapy sessions were well spread out at 1-2 week intervals. | 5 | 5 |
| Therapy sessions were tailored to my needs | Talks about therapy sessions generally being tailored to their needs (positive statement). Code specific tailoring for sleep behaviour change and emotion regulation tailoring separately. | 14 | 37 |
| Timing of participation | Timing of participation in trial was a facilitator to engagement. Suggestion that if they had taken part at a different time i.e. term time, their busy schedule would have been a barrier. | 1 | 1 |
| Tracker - easy to use | Statement that sleep trackers (Actiwatches) were easy to use. | 2 | 2 |
| User-friendly digital content | Specific references to the online programme being 'user-friendly', 'easy to follow', 'easy to use'. Some overlap with code 'overall programme was straightforward'. The latter refers to the whole package, rather than just to digital content. | 9 | 15 |
| Validation or reassurance | Provision of validation or reassurance to encourage engagement. | 5 | 12 |
| Valued human interaction | Really liked the therapist contact element of the programme. | 5 | 11 |
| Well paced programme | Talks about the programme flowing well from week to week. Relates to programme length, although this is different from the sense of 'pace'. | 5 | 7 |
| Willingness and readiness to change (internal motivation) | Participant getting what they want/need from the programme if they took it seriously and engaged with it. Internal motivation and readiness to change facilitated better outcomes. | 1 | 1 |
| **Overall experience** [FRAMEWORK CODE/ TOP-LEVEL] | Talking about their overall experience of the intervention and outcomes. Positive/ negative/ neutral. Also code reflections on skills they have learnt as a result of the programme (skills-based outcomes) | 0 | 0 |
| Colleagues were curious | Talks about colleagues being curious about involvement in study. | 2 | 2 |
| Confidence moving forwards | Talks about being able to notice triggers/ red flags themselves. Links to 'planning for insomnia returning' content. Include confidence in both insomnia and MH management. | 7 | 12 |
| Curious about tracker results | Refers to curiosity about what the tracker would show - potentially would have liked access to these results, but understood why not. Neutral statement. | 4 | 9 |
| Employers should focus on sleep | Talks about how employers should focus more on programmes to improve sleep as opposed to general wellbeing. | 1 | 2 |
| Encouraged self-care | Enabled participant to take time for themselves. | 5 | 18 |
| Enjoyable programme | Talks about the programme being enjoyable or 'nothing they didn't enjoy'. | 6 | 12 |
| Experience - mixed feelings | Mixed feelings - liked online content, disliked therapist sessions. Poor rapport, lack of confidence in therapist. | 1 | 1 |
| 'Fresh during the day' | In vivo code - talks about feeling more resilient, fresh and energetic during the daytime, as a result of being on programme. | 9 | 17 |
| Good if you haven't tried similar before | Talks about programme potentially being good if SRT had not been tried before. | 1 | 2 |
| Guilt - gaining something from COVID-19 | Feels guilty that they have gained something positive out of COVID-19. Statement of a mixed feeling about outcomes. | 1 | 1 |
| I can manage my sleep better than before | References to being able to manage/ cope/ deal with sleep issues better than they were able to prior to taking part in the programme. Include continuing to practice techniques. | 13 | 27 |
| I was actually telling everyone about it | In vivo code; code all that refer to sharing advice and learning with family and friends or colleagues. | 4 | 6 |
| I'm not sure its 'deep sleep' | Outcomes. Not certain that they are getting deep sleep. Linked to Fitbit use. | 1 | 4 |
| Importance of focusing on sleep | Reiteration or realisation of the need to focus on sleep/ why focusing on sleep is important. | 5 | 7 |
| Improved concentration | Notes improved concentration as an outcome relative to improved sleep quality. | 1 | 1 |
| Improved insomnia symptoms | References to improvement in clinical/ sub insomnia symptoms. Includes reduced sleep onset time, reduced waking, increased hours sleeping. Code 'improved my sleep quality' separately. | 12 | 32 |
| Improved my sleep quality | References to improvements in sleep in general, including references to quality of sleep. Subjective evaluation of sleep quality. | 14 | 23 |
| Improved sleep hygiene | Marked improvements in sleep hygiene e.g. caffeine intake. | 1 | 1 |
| Improving work-life balance | Talks about improvements to work-life balance as a result of programme participation. | 9 | 13 |
| Increased optimism about sleep | Talks about feeling more 'optimistic' about the future of her sleep behaviour after finishing the programme. | 1 | 4 |
| Mental health signposting - therapists' suppport | Talks about the signposting part of the programme being important, with reference to therapist support with this being key to engaging with further mental health treatment. | 1 | 8 |
| More time to do other things | Talks about feeling as though they have more time in the day to fit everything in, especially on the weekends. Better time management as a result of routine sleep schedule. Positive talk. | 3 | 6 |
| No change experienced | No change experienced. No outcomes, they are the same. | 1 | 1 |
| Open it up to everyone | Statement that this should be an openly accessible programme to all. | 2 | 2 |
| Outcome measures - wording - difficult | Found the wording of scale questionnaire measures made it difficult to respond, difficult to select answer. | 1 | 1 |
| Physical improvements | Noted improvements to physical health. | 1 | 1 |
| Positive experience - general | Talks about overall experience of programme being positive. Or good/great/excellent. Similar to code 'enjoyable programme', but code specific use of word 'enjoy' separately. | 16 | 50 |
| Practising relaxation and stress management | Talks about practising relaxation techniques they learnt in the programme and how this has been helpful to reduce stress/ worry. Include vague references to reducing stress or coping with stress. | 9 | 17 |
| Programme was straightforward | References to whole programme being 'straightforward', 'easy to follow', and other synonyms. Code 'user-friendly' digital content separately. | 11 | 21 |
| Psychoeducation - understanding SCT principles | Client gained understanding of the relationship between being in bed verses being asleep, and draws on this as a key principle. Acknowledgement that lying in bed awake does not improve sleep quality. | 11 | 16 |
| Reduced napping | Improvements to sleep quality and family life due to stopping napping. | 3 | 8 |
| Refilled an 'empty cup' | in vivo code - relates to being in a better place mentally - a better position to be able to help others (family/friends etc), following participation in study. | 1 | 3 |
| Shared techniques with colleagues | Explains that they shared techniques from programme with colleagues. | 2 | 6 |
| Signposting was valuable | Signposting to other/further information referred to as helpful or valuable. Specific references to signposting in online material. Code help with signposting via therapist under 'Mental health signposting - therapists' support'. | 1 | 1 |
| Sleep as an access point for other therapy | Talks about using sleep/ SLEEP programme as an access point or prompt to accessing other therapies (CBT, counselling etc.) | 5 | 9 |
| Sleep restriction was worth it | Talks about sleep restriction (SRT) being worth the challenge/ initial struggle/ tiredness. | 4 | 4 |
| Sleep should be a focus in schools | States that sleep education should be more of a focus in schools. | 1 | 1 |
| Sleep went downhill after trial | Talks about experience after trial - sleep problems returning once the sessions had finished. Routine not maintained going forward. Various reasons for this - code reason in addition. | 4 | 6 |
| Small improvements make a difference (sleep) | Talk about small improvements making a significant difference to individual's perception of how good their sleep is i.e. 'to me a small difference makes all the difference.' May be more evident in clients with worse sleep to begin with. | 3 | 4 |
| Spillover effect (behavioural mechanism) | Spillover effect, when changes in one domain show positive effects, there is a tendency to apply into other areas. For example improved sleep = improved mood/ more positive general outlook. | 16 | 49 |
| Still searching for the cause of insomnia | Questioning whether there is a reason why they're not sleeping - has not uncovered this during programme. Has not gained any further understanding or answers about the cause of insomnia. | 1 | 2 |
| Surprisingly positive outcomes | Use of e.g.,“actually”, suggesting they didn’t expect to make any improvement, but did. Sense of surprise. | 8 | 25 |
| Would have liked more therapist sessions | Statement that the participant would have liked more (greater number) of therapist sessions. | 5 | 13 |
